# Tumour prothymosin alpha content, a potential prognostic marker for primary breast cancer

**DOI:** 10.1054/bjoc.1999.0968

**Published:** 2000-01-18

**Authors:** C Magdalena, F Dominguez, L Loidi, J L Puente

**Affiliations:** Departamento de Cirugia, Hospital Xeral de Galicia, Spain; Departamento de Fisiologia, Laboratorio de Neurociencia ‘Ramon Dominguez’ and Unidad de Medicina Molecular, Facultad de Medicina, Santigao de Compostela, 15705, Spain

**Keywords:** thymosin alpha 1 radioimmunoassay, tumour proliferation marker, overall mortality

## Abstract

In a previous report we suggested that the estimation of prothymosin α (PTA) levels in primary breast tumours might be used to identify breast cancer patients at high risk for distant metastasis (Dominguez F et al (1993) *Eur J Cancer***29A**: 893–897). Here the role of tumour PTA levels as predictor was investigated with respect to both disease-free survival (DFS) and survival. Tumours were obtained from a series of 210 consecutive female patients with ductal carcinoma who underwent surgery at the Hospital Xeral de Galicia (Santiago de Compostela, Spain). Characteristics including PTA tumour levels, number of positive axillary nodes, patient's age at surgery and tumour histological grade were significantly associated with DFS and survival, as determined by univariate analysis. Patients with tumours with low or moderate PTA levels demonstrated a statistically decreased rate of tumour recurrence and a statistically significant increased overall survival in comparison with those whose tumours had high PTA levels. Patient's relative risk of dying was 2.1 times greater for tumours with high PTA levels than for those tumours with low or moderate PTA levels. In conclusion, these data support the hypothesis that tumour high PTA levels is associated with a worse outcome. © 2000 Cancer Research Campaign


*British Journal of Cancer* (2000) **82**(3), 584–590
© 2000 Cancer Research Campaign
DOI: 10.1054/ bjoc.1999.0968, available online at http://www.idealibrary.com on IDEAL®

### Tumour prothymosin alpha content, a potential prognostic marker for primary breast cancer

**C Magdalena[1], F Dominguez[2], L Loidi[2] and JL Puente[1]**

[1]Departamento de Cirugia, Hospital Xeral de Galicia, Spain; [2]Departamento de Fisiologia, Laboratorio de Neurociencia 'Ramon Dominguez' and Unidad de Medicina Molecular, Facultad de Medicina, 15705 Santigao de Compostela, Spain

**Summary** In a previous report we suggested that the estimation of prothymosin α (PTA) levels in primary breast tumours might be used to identify breast cancer patients at high risk for distant metastasis (Dominguez F et al (1993) *Eur J Cancer* **29A:** 893–897). Here the role of tumour PTA levels as predictor was investigated with respect to both disease-free survival (DFS) and survival. Tumours were obtained from a series of 210 consecutive female patients with ductal carcinoma who underwent surgery at the Hospital Xeral de Galicia (Santiago de Compostela, Spain). Characteristics including PTA tumour levels, number of positive axillary nodes, patient's age at surgery and tumour histological grade were significantly associated with DFS and survival, as determined by univariate analysis. Patients with tumours with low or moderate PTA levels demonstrated a statistically decreased rate of tumour recurrence and a statistically significant increased overall survival in comparison with those whose tumours had high PTA levels. Patient's relative risk of dying was 2.1 times greater for tumours with high PTA levels than for those tumours with low or moderate PTA levels. In conclusion, these data support the hypothesis that tumour high PTA levels is associated with a worse outcome. © 2000 Cancer Research Campaign

**Keywords:** thymosin alpha 1 radioimmunoassay; tumour proliferation marker; overall mortality

Prothymosin α (PTA) is a small highly acidic nuclear protein related to normal cell proliferation (Haritos et al, 1984; Eschenfeldt and Berger, 1986; Goodall et al, 1986; Gomez et al, 1989; Conteas et al, 1990; Zalvide et al, 1992). Immunohistochemical studies have shown that PTA is expressed in proliferating but not in quiescent cells in all tissues studied so far (Conteas et al, 1990; Fraga et al, 1993; Gallego et al, 1992; Garcia et al, 1994; Roson et al, 1990*a*, 1990*b*, 1993). PTA is expressed in various human tumours of different origins (reviewed in Dominguez et al, 1997), supporting the idea that PTA expression is required for tumour growth. Certainly, when PTA expression is blocked the proliferation of myeloma cells is inhibited (Sburlati et al, 1991). Non-transformed NIH 3T3 cells shows a similar behaviour (Rodriguez et al, 1998), indicating that PTA must play an essential role in normal proliferation that is preserved in tumorous cells. Moreover, when PTA is overexpressed the G1 phase of the cell cycle is shortened (Rodriguez et al, 1998) as it also happens with other factors that play a major role in cell proliferation. This finding indicates that PTA must be a limiting factor necessary for the progression of the cell cycle. On the other hand, the analysis of the intracellular signalling pathways governing PTA expression point to the fact that PTA expression is an event occurring downstream in several mitogenic pathways. PTA expression seems to be under the direct control of MYC (Eilers et al, 1991); however, other reports did not confirm this finding (Mol et al, 1995). Vareli et al (1996) have found that the transcription factor E2F activates a reporter gene under the PTA promoter, indicating that E2F could directly control PTA expression. In mammary tumours arising in *c-myc*, *c-neu* and *v-ras* transgenic mice we found that there is a differential regulation of PTA with respect to other putative *c-myc* target genes, indicating that regulation of PTA in these tumours is complex and depends on more than a single activated oncogene (Loidi et al, 1999).

PTA physiological function remains unknown. It is a nuclear protein (Gomez and Segade, 1988; Roson et al, 1990*a*) that is present throughout the cell cycle (Zalvide et al, 1992); it has been shown that it binds histones in vitro (Papamarcaki and Tsolas, 1994; Diaz et al, 1996) and has been proposed to affect the chromatin state (Gomez-Marquez et al, 1998). Recently, we have found that PTA modulates the activity of calcium/calmodulin-dependent kinases (CaM-kinase) II and III in a cell cycle-dependent manner (Vega et al, 1998). These enzymes play various roles during the cell cycle. CaM-kinase III is necessary for the phosphorylation of EF-2 during mitosis (Ryazanov, 1987; Celis et al, 1990) and CaM-kinase II affects the $G_1$ progression as well as the transition from $G_2$ to mitosis (Lu and Means, 1993).

We had previously shown in 71 patients with invasive ductal carcinomas, that PTA levels as assayed by a radioimmunoassay that detects thymosin $\alpha_1$, the $NH_2$-terminal fragment of PTA, were significantly greater in tumour samples than in normal breast tissue. PTA levels were correlated with: (a) the number of positive axillary lymph nodes, and (b) the percentage of tumour cells in the S phase plus in the G2/M phase as assessed by flow cytometry. Of special relevance was to find that PTA levels might be used to identify patients at high risk for distant metastasis (Dominguez et al, 1993). Recently, others independently confirmed these findings (Tsitsilonis et al, 1998). In the present report, the role of tumour PTA content as predictor was investigated with respect to both disease-free survival and overall survival.

*Received 3 March 1999*
*Revised 22 July 1999*
*Accepted 3 August 1999*

*Correspondence to:* F Dominguez

**Table 1** Patient and tumour characteristics

| | PTA levels [No. (%)[a]] | | | |
|---|---|---|---|---|
| | Low | Moderate | High | |
| No. of patients | 210 | | | |
| Median follow-up, months | | | | |
| Median | 36 | | | |
| Range | 2–106 | | | |
| Tumour size, cm | | | | |
| ≤2 | 15 (18) | 49 (59) | 19 (23) | |
| >2 | 18 (14) | 73 (58) | 35 (28) | 0.336[b] |
| No. of positive lymph nodes | | | | |
| None | 19 (56) | 44 (37) | 17 (31) | |
| 1–3 | 11 (32) | 37 (31) | 13 (24) | |
| 4–9 | 2 (6) | 22 (18) | 14 (26) | |
| ≥10 | 2 (6) | 17 (14) | 10 (18) | 0.004[b] |
| Histological grade | | | | |
| Well differentiated | 10 (32) | 27 (23) | 5 (10) | |
| Moderately differentiated | 15 (48) | 54 (46) | 15 (29) | |
| Poorly differentiated | 6 (19) | 37 (31) | 32 (61) | 0.000[b] |
| DNA ploidy | | | | |
| Diploid | 4 (44) | 14 (28) | 1 (4) | |
| Aneuploid | 5 (56) | 36 (72) | 21 (95) | 0.008[b] |
| Age at surgery, (years) | | | | |
| ≤ 39 | 1 (3) | 9 (7) | 7 (13) | |
| 40–49 | 4 (12) | 26 (22) | 11 (20) | |
| ≥ 50 | 28 (85) | 85 (71) | 36 (67) | 0.049[b] |

[a]Unless otherwise specified, values in columns = number of subjects (%). [b]Mantel–Haenszel test for linear association.

### PATIENTS'. MATERIALS AND METHODS

### Patients

High PTA levels in tumours have been suggested to be a predictor of a poor prognosis (Dominguez et al, 1993). A prospective study was designed to test this hypothesis. Tumours were obtained from a series of 210 consecutive female patients with invasive ductal carcinoma who underwent definitive surgery at the Hospital General de Galicia (Santiago de Compostela, Spain) since 1989. Patients with a second malignant neoplasm were excluded.

### Specimen analysis

Each tissue block was sectioned for histological analysis as previously described (Dominguez et al, 1993). Nodal involvement was histologically determined. Tumour grading was done according to the Bloom and Richardson method as described by Freedman et al (1979). Ploidy analysis was done by DNA flow cytometry. Tumour samples were processed as previously described (Dominguez et al, 1993).

### PTA radioimmunoassay

PTA radioimmunoassay (RIA) was done as previously published (Dominguez et al, 1993). Briefly, slices (about 200 mg) of tumour tissues obtained during surgery were homogenized with a Polytron homogenizer in phosphate-buffered saline (PBS)-EDTA, centrifuged at 14 000 ***g*** for 15 min and the supernatant analysed for PTA and protein. The radiolabelled ligand was $I^{125}$-$Tyr^{0}$-thymosin $\alpha_1$ and synthetic thymosin $\alpha_1$ was employed to standardize the assay. Tissue homogenates were prepared at room temperature in order to convert tissue PTA to thymosin $\alpha_1$; therefore, the results are expressed as thymosin $\alpha_1$ equivalents (Dominguez et al, 1993).

### Patient outcome end points

The role of tumour PTA levels as predictor was investigated with respect to both disease-free survival (DFS) and survival; as secondary end point, we also examined distant disease-free survival (DDFS). DFS was defined as the time from surgery prior to recurrent breast cancer or death from any cause. Survival was defined as the time from surgery to death from any cause. DDFS was defined as the time from surgery to tumour recurrence at distant sites.

### Statistical analysis

Logistic regression analysis, Kaplan–Meier survival analysis and the Cox regression model were used. All data were analysed with the use of the SPSS statistical package (SPSS Inc., Chicago, IL USA).

### RESULTS

### Patient characteristics

Table 1 summarizes the clinical and biological data for all 210 patients enroled in this study. Patients were divided in three groups according to tumour PTA content as follows: low level, PTA levels ranging from 0 to 20 ng $mg^{-1}$ of protein (34 patients); moderate

© 2000 Cancer Research Campaign

**Table 2** Number of patients exposed to risk and hazard rate

| | | Survival | | DFS | | DDFS | |
|---|---|---|---|---|---|---|---|
| | | **No.** | **hr ± s.e.m.** | **No.** | **hr ± s.e.m.** | **No.** | **hr ± s.e.m.** |
| PTA Levels | | | | | | | |
| Low | *Year* | | | | | | |
| | 0 | 33.0 | 0.0000 ± 0.0000 | 33.0 | 0.0000 ± 0.0000 | 33.0 | 0.0000 ± 0.0000 |
| | 1 | 32.0 | 0.0000 ± 0.000 | 32.0 | 0.0054 ± 0.0038 | 32.0 | 0.0026 ± 0.0026 |
| | 2 | 26.5 | 0.0000 ± 0.000 | 25.0 | 0.0034 ± 0.0034 | 26.0 | 0.0033 ± 0.0033 |
| | 3 | 18.5 | 0.0000 ± 0.000 | 17.0 | 0.0104 ± 0.0074 | 18.0 | 0.0048 ± 0.0048 |
| | 4 | 11.5 | 0.0000 ± 0.000 | 8.5 | 0.0000 ± 0.0000 | 11.0 | 0.0000 ± 0.0000 |
| | 5 | 4.5 | 0.0000 ± 0.000 | 3.0 | 0.0000 ± 0.0000 | 5.0 | 0.0185 ± 0.0184 |
| Moderate | 0 | 121.0 | 0.0014 ± 0.0010 | 121.0 | 0.0064 ± 0.0021 | 121.0 | 0.0028 ± 0.0014 |
| | 1 | 114 | 0.0045 ± 0.0018 | 107.0 | 0.0162 ± 0.0037 | 112.0 | 0.0078 ± 0.0025 |
| | 2 | 90 | 0.0048 ± 0.0021 | 72.0 | 0.0085 ± 0.0032 | 84.0 | 0.0030 ± 0.0017 |
| | 3 | 55 | 0.0015 ± 0.0015 | 39.5 | 0.0066 ± 0.0038 | 53.0 | 0.0083 ± 0.0037 |
| | 4 | 31 | 0.0055 ± 0.0039 | 18.5 | 0.0046 ± 0.0046 | 27.5 | 0.0063 ± 0.0044 |
| | 5 | 15 | 0.0057 ± 0.0057 | 8.5 | 0.0000 ± 0.0000 | 12.5 | 0.0000 ± 0.0000 |
| High | 0 | 53.5 | 0.0048 ± 0028 | 54.0 | 0.0208 ± 0.0060 | 54.0 | 0.0170 ± 0.0053 |
| | 1 | 48.5 | 0.0213 ± 0.0064 | 41.0 | 0.0156 ± 0.0059 | 43.0 | 0.0125 ± 0.0051 |
| | 2 | 30.0 | 0.0119 ± 0.0059 | 27.5 | 0.0243 ± 0.0091 | 30.0 | 0.0220 ± 0.0082 |
| | 3 | 16.0 | 0.0238 ± 0.0118 | 11.5 | 0.0250 ± 0.0143 | 13.0 | 0.0217 ± 0.0124 |
| | 4 | 6.5 | 0.0500 ± 0.0275 | 4.0 | 0.1000 ± 0.0462 | 4.0 | 0.0556 ± 0.0370 |
| | 5 | 2.0 | 0.1667 ± 0.0000 | | | | |

No., number of patients at risk; hr = hazard rate.

**Table 3** Univariate analysis of variables associated with DFS and survival

| | No. of patients[a] | DFS | | | Survival | | |
|---|---|---|---|---|---|---|---|
| | | **RR[b]** | **95% CI** | ***P*** [c] | **RR[b]** | **95% CI** | ***P*** [c] |
| PTA levels | 209 | 1.61 | 1.28–2.03 | 0.0001 | 2.63 | 1.93–3.59 | 0.000 |
| Tumour size (cm) | 208 | 1.13 | 0.89–1.43 | 0.3045 | 1.48 | 1.05–2.09 | 0.022 |
| Positive lymph nodes | 208 | 1.40 | 1.08–1.81 | 0.0090 | 2.10 | 1.36–3.24 | 0.000 |
| Histological grade | 200 | 1.50 | 1.19–1.88 | 0.0024 | 1.96 | 1.44–2.67 | 0.000 |
| Age at surgery (years) | 206 | 0.76 | 0.60–0.96 | 0.0241 | 0.658 | 0.48–0.88 | 0.006 |

[a]Number of patients indicates number with complete data. The numbers of patients' tumours analysed are not identical for all categories because of incomplete or unavailable data on some cases. [b]The use of continuous variables provides lower *P*-values, however, RR compares two categories for each variable. Therefore, for continuous variable, we selected specific values to illustrate how to interpret the RR for the following variables: PTA levels (high vs low and moderate), size (> 2 cm vs ≤ 2 cm), lymph nodes (none vs ≥ 1), histological grade (poorly differentiated vs well and moderately differentiated), age (>50 years vs ≤ 50 years). [c]*P*-values are from the Cox proportional hazards model with the use of the Walds' test.

level, PTA levels ranging from 20 to 100 ng $mg^{-1}$ of protein (122 patients); and high level, PTA levels greater than 100 ng $mg^{-1}$ of protein (54 patients). A significant Pearson correlation was found between PTA levels and number of positive lymph nodes (two-sided $P < 0.05$), histological grade (two-sided $P < 0.01$), DNA ploidy (two-sided $P < 0.01$) and patient's age at surgery ($P = 0.05$). By means of a logistic regression model relating PTA levels (high vs low and moderate) with all co-variates, we found that the histological grade was significantly associated with PTA high levels ($P < 0.001$). On the other hand, PTA low levels (low vs moderate and high) were significantly associated with the absence of positive nodes ($P < 0.03$) and with the histological grade ($P < 0.03$).

### Analysis of outcomes

Table 2 shows estimates of the number of patients at risk and the hazard rate for the first 5 years. Figure 1 shows Kaplan–Meier plots for overall survival, DFS and DDFS for PTA levels. Patients with low levels of PTA showed a superior behaviour to patients with moderate or high PTA levels on the basis of each end point. Characteristics including PTA tumour levels, number of positive axillary nodes, patient's age at surgery and tumour histological grade were significantly associated with DFS and survival, as determined by univariate analysis (Table 3). The same variables plus tumour size were significantly associated with overall survival (Table 3). The multivariate analysis of DFS and survival is shown in Table 4. The effect of PTA levels, number of positive lymph nodes and the histological grade is significant using the multivariate model while tumour size and the age of the patient loses the significance found in the univariate analysis.

As seen in Table 4 the risk of patients with tumours with high PTA levels relative to tumours with low to moderate PTA levels is high for both DFS and survival. In Figure 2, the data is stratified according to other covariates of clinical relevance. The risk of patients with tumours with high PTA levels relative to tumours with low or moderate PTA levels is apparent among patients with

 *© 2000 Cancer Research Campaign*

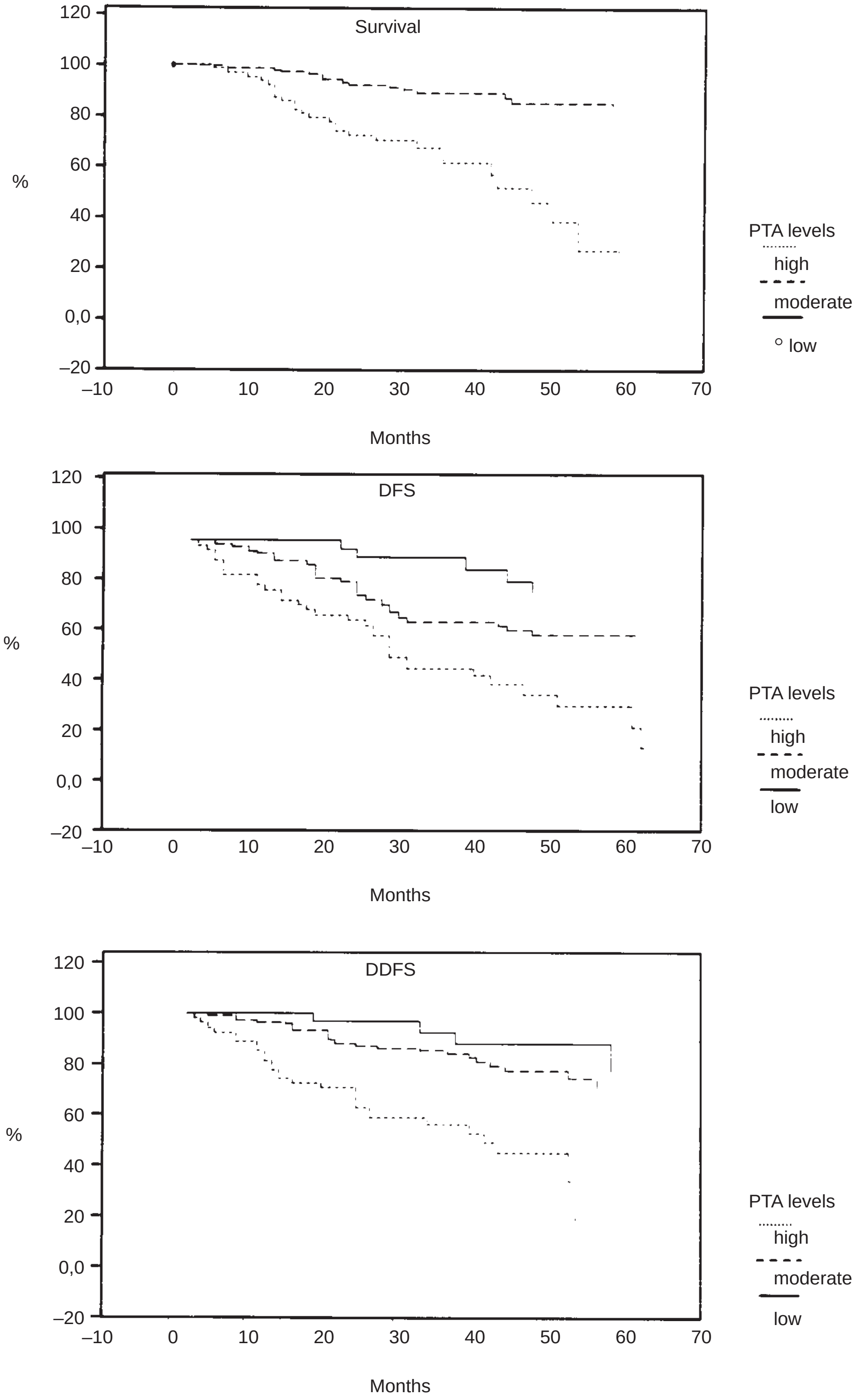

**Figure 1** Plot of estimated survival, DFS and DDFS function across PTA levels groups. Patients were divided in three groups according to tumour PTA content as follows: low level, PTA levels ranging from 0 to 20 ng $mg^{-1}$ of protein; moderate level, PTA levels ranging from 20 to 100 ng $mg^{-1}$ of protein; and high level, PTA levels greater than 100 ng $mg^{-1}$ of protein. All patients with low PTA levels were alive during the period of observation negative axillary lymph nodes. This is also true for other covariates shown in Figure 2 but the number of patients in each stratum is low; therefore, present data should be further validated. The central hypothesis of this investigation was to see if patients with tumours with high proliferating activity, as seen by estimating tumour PTA levels, have a worse outcome. Bonferronis joint multivariate analysis was used to test the effect of high PTA levels on patient's outcome using as primary end point survival, DFS and DDFS and the following co-variates: tumour size (> 2 cm vs ≤2 cm), positive lymph nodes (none vs ≥ 1), histological grade (poorly differentiated vs well and moderately differentiated), and age at surgery (> 50 years vs ≤ 50 years). The hypothesis was significant for the three end points, survival ($P$ = 0.000), DFS ($P$ = 0.04149) and DDFS ($P$ = 0.0001).

*© 2000 Cancer Research Campaign* 

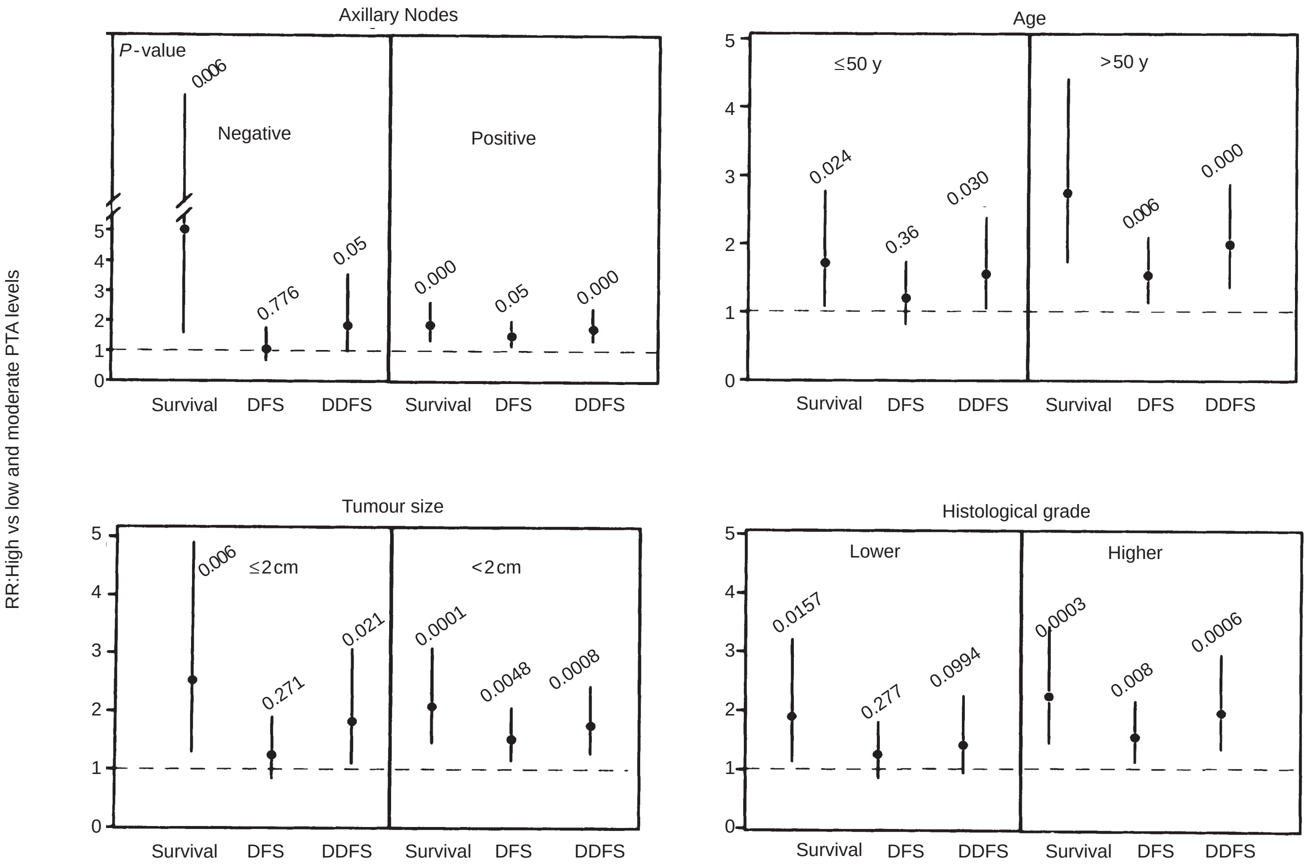

**Figure 2** Risks of patients with high PTA levels tumours (●) relative to those of patients with tumours with low or moderate PTA levels. Risk of patients with tumours with low or moderate PTA levels is considered to be 1 (dotted line). Relative risks of failure (RRs) and *P*-values shown on each plot are adjusted (Cox model with the use of the Wald's test) for patient's lymph node status, age at the time of surgery, tumour size and the histological grade. Bars indicate 95% confidence intervals. All *P*-values are two-sided. DFS = disease-free survival; DDFS = distant disease-free survival

**Table 4** Multivariate Cox proportional analyses of variables associated with DSF and survival

| | DFS | | | Survival | | |
|---|---|---|---|---|---|---|
| **Variable** | **RR[a]** | **95 % CI** | ***P*[b]** | **RR[a]** | **95 % CI** | ***P*[b]** |
| PTA levels | 1.42 | 1.12–1.81 | 0.003 | 2.10 | 1.52–2.90 | 0.000 |
| Tumour size, cm | 1.04 | 0.82–1.32 | 0.721 | 1.25 | 0.87–1.78 | 0.219 |
| Positive lymph nodes | 1.31 | 1.01–1.70 | 0.038 | 1.78 | 1.15–2.75 | 0.030 |
| Histological grade | 1.34 | 1.06–1.69 | 0.014 | 1.44 | 1.03–2.01 | 0.007 |
| Age at surgery, (years) | 0.84 | 0.67–1.07 | 0.174 | 0.84 | 0.61–1.15 | 0.287 |

[a]The use of continuous variables provides lower *P*-values, however, RR compares two categories for each variable. Therefore, we selected specific values to illustrate how to interpret the RR for the following variables: PTA levels (high versus low and moderate), size (> 2 cm vs ≤ 2 cm), lymph nodes (none vs ≥ 1), histological grade (poorly differentiated vs well and moderately differentiated), age (>50 years vs ≤ 50 years). [b]*P*-values are from the Cox proportional hazards model with the use of the Walds' test.

### DISCUSSION

The goal of the current study was to determine if tumours with high PTA levels were associated with a worse outcome. We have analysed the overall survival of patients with breast tumours that underwent definitive surgery since the beginning of this study in 1989. PTA levels were assayed by a radioimmunoassay that detects thymosin $\alpha_1$, the $NH_2$-terminal fragment of PTA (Dominguez et al, 1993). We present new evidences indicating that estimation of PTA levels in the tumour is a potential prognostic marker for primary breast cancer. A statistically significant association between PTA levels and other relevant clinical factors as the number of positive lymph nodes, the histological grade, DNA ploidy and the age at surgery was observed. Patients with tumours with low or moderate PTA levels demonstrated a statistically significant decreased rate of tumour recurrence and a statistically significant increased overall survival in comparison with those patients whose tumours had high PTA levels. Multivariate Cox

 *© 2000 Cancer Research Campaign*

proportional analyses showed that those patients with high PTA levels have 2.1 times the risk of dying than patients with low or moderate PTA levels. Moreover, patients with high PTA levels had a greater risk (1.42 times) of regional or distant tumour recurrence than patients with low or moderate PTA levels. However, these numbers must be taken with caution since, as always, the model will fit the sample from which it is estimated better than it will fit the population from which the sample is selected. Bonferronis joint multivariate analysis was used to test the effect of high PTA levels on patient's outcome using as primary end point survival, DFS and DDFS. PTA levels had significant effect on the three end points. The fact that the three end points were significantly affected provides a stronger support to the hypothesis that patients with tumours with high PTA levels have a worse outcome. These findings are not surprising since cell proliferation indices have been shown to be prognosis-related (Freedman et al, 1979; Sigurdsson et al, 1990). By means of immunohistochemical techniques we found that PTA and the proliferating cell nuclear antigen (PCNA) had a similar pattern of expression both in normal tissues (Roson et al, 1993) and hepatocellular carcinomas (Fraga et al, 1993). PTA is expressed in the late G1, S, G2 and M phases of the cell cycle (Zalvide et al, 1992). Interestingly, the fraction of cells in S and G2 phases is more closely associated with the clinical outcome than the S phase fraction alone (Witzig et al, 1994). Recently, Rudolph et al, using the proliferation marker Ki-S2 concluded that the prognosis is best indicated by the percentage of cells in S through M phases of the cell cycle (Rudolph et al, 1999). In agreement with this we found in breast tumours that PTA expression was correlated with the percentage of cells in S and G2/M phases fraction but not with the S phase alone (Dominguez et al, 1993). Here we presented the estimation of tumour proliferation rate as assessed using a RIA for PTA. PTA RIA provides an objective measure of tumour proliferation activity.

A major trend in the biologic research in breast cancer is focused in factors that predict benefit from therapy. At present, it is not possible to identify those patients who are cured without adjuvant chemotherapy or those patients who will relapse in spite of adjuvant chemotherapy, with resistant disease (Powles, 1997). Tumour PTA content was investigated as predictor with respect to DFS, DDFS and overall survival. Patients received surgical, radiation and other adjuvant therapies following an established pattern, irrespectively of tumour PTA content. Patients with tumours with high PTA levels had a worst outcome irrespective of the adjuvant therapy received. On the other hand, tumours with low levels of PTA had a good prognosis. Further studies with a larger series of patients are now needed to establish the role of tumour PTA as a predictor of the therapeutic response. However, it is interesting to point out that PTA antisense oligonucleotides that blocks PTA expression promoted apoptosis in HL60 cells (Rodriguez et al, 1999). If this finding is confirmed it is tempting to speculate that high PTA levels could have a protective effect against apoptosis induced by chemotherapeutic agents.

In summary, this paper reaffirms two major points in breast cancer research: first, the importance that tumour's proliferation rates have in patient's outcome and, second, the benefit of using quantitative assays to estimate proliferation rates. We propose that PTA content could be used as a predictor of the potential malignancy of breast tumours that might help to identify patients at high risk of dying. Our study represents a preliminary attempt to correlate tumour PTA levels and overall mortality in breast cancers.

### ACKNOWLEDGEMENTS

Supported in part by FISS grants (Fondo de Investigaciones Sanitarias de la Seguridad Social 92/0649 to JLP, 93/0475 to FD) and XUGA grants (Xunta de Galicia 94/20818B to JLP, and Conselleria de Sanidade e Servicios Sociais. Dirección Xeral de Saude Pública. Programa de Screening de Cancer de Mama to FD).

### REFERENCES

Celis JE, Madsen P and Ryazanov AG (1990) Increased phosphorylation of elongation factor 2 during mitosis in transformed human amnion cells correlates with a decreased rate of protein synthesis. *Proc Natl Acad Sci USA* **87**: 4231–4235

Conteas CN, Mutchnick MG, Palmer KC, Weller FE, Luk GD, Naylor PH, Erdos MR, Goldstein AL, Panneerselvam C and Horecker BL (1990) Cellular levels of thymosin immunoreactive peptides are linked to proliferative events: evidence for a nuclear site of action. *Proc Natl Acad Sci USA* **87**: 3269–3273

Diaz JC, Perez EA, Covelo G and Freire M (1996) Prothymosin alpha binds histones in vitro and shows activity in nucleosome assembly assay. *Biochim Biophys Acta* **1296**: 219–227

Dominguez F, Gallego R, Roson E, Loidi L and Garcia-Caballero T (1997) Prothymosin alpha distribution in normal and tumoral tissues. *Int J Thymology* **5**: 474–476

Dominguez F, Magdalena C, Cancio E, Roson E, Paredes J, Loidi L, Zalvide J, Fraga M, Forteza J, Regueiro BJ and et al (1993) Tissue concentrations of prothymosin alpha: a novel proliferation index of primary breast cancer. *Eur J Cancer* **29A**: 893–897

Eilers M, Schirm S and Bishop JM (1991) The MYC protein activates transcription of the alpha-prothymosin gene. *EMBO J* **10**: 133–141

Eschenfeldt WH and Berger SL (1986) The human prothymosin alpha gene is polymorphic and induced upon growth stimulation: evidence using a cloned cDNA. *Proc Natl Acad Sci USA* **83**: 9403–9407

Fraga M, Garcia CT, Dominguez F, Perez BE, Beiras A and Forteza J (1993) Immunohistochemical location of prothymosin alpha in regenerating human hepatocytes and hepatocellular carcinomas. *Virchows Arch A Pathol Anat Histopathol* **423**: 449–452

Freedman LS, Edwards DN, McConnell EM and Downhay DY (1979) Histological grade and other prognosis factors in relation to survival of patients with breast cancer. *Br J Cancer* **40**: 44–55

Gallego R, Roson E, Garcia CT, Fraga M, Forteza J, Dominguez F and Beiras A (1992) Prothymosin alpha expression in lymph nodes and tonsils: an optical and ultrastructural study. *Acta Anat Basel* **143**: 219–222

Garcia CT, Dominguez F, Roson E, Gallego R, Zalvide J, Forteza J and Beiras A (1994) Distribution of prothymosin alpha in rat and human adrenal cortex. *Anat Rec* **239**: 88–94

Gomez-Marquez J and Rodriguez P (1998) Prothymosin alpha is a chromatin-remodelling protein in mammalian cells. *Biochem J* **333**: 1–3

Gomez MJ and Segade F (1988) Prothymosin alpha is a nuclear protein. *FEBS Lett* **226**: 217–219

Gomez MJ, Segade F, Dosil M, Pichel JG, Bustelo XR and Freire M (1989) The expression of prothymosin alpha gene in T lymphocytes and leukemic lymphoid cells is tied to lymphocyte proliferation. *J Biol Chem* **264**: 8451–8454

Goodall GJ, Dominguez F and Horecker BL (1986) Molecular cloning of cDNA for human prothymosin alpha. *Proc Natl Acad Sci USA* **83**: 8926–8928

Haritos AA, Goodall GJ and Horecker BL (1984). Prothymosin alpha: isolation and properties of the major immunoreactive form of thymosin alpha 1 in rat thymus. *Proc Natl Acad Sci USA* **81**: 1008–1011

Loidi L, Garcia-Caballero T, Vidal A, Zalvide J, Gallego R and Dominguez F (1999) Complex regulation of prothymosin alpha in mammary tumors arising in transgenic mice. *Life Sci* **64**: 2125–2133

Lu KP and Means AR (1993) Regulation of the cell cycle by calcium and calmodulin. *Endocr Rev* **14**: 40–58

Mol PC, Wang RH, Batey DW, Lee LA, Dang CV and Berger SL (1995) Do products of the myc proto-oncogene play a role in transcriptional regulation of the prothymosin alpha gene? *Mol Cell Biol* **15**: 6999–7009

Papamarcaki T and Tsolas O (1994) Prothymosin alpha binds to histone H1 in vitro. *FEBS Lett* **345**: 71–75

Powles TJ (1997) Adjuvant therapy for early breast cancer: a time to refine. *J Natl Cancer Inst* **89**: 1652–1654

© 2000 Cancer Research Campaign

Rodriguez P, Viñuela JE, Alvarez-Fernandez L, Buceta M, Vidal A, Dominguez F, Gomez-Marquez J (1998) Overexpression of prothymosin alpha accelerates proliferation and retards differentiation in HL-60 cells. *Biochem J* **331**: 753–761

Rodriguez P, Vinuela JE, Alvarez-Fernandez L and Gomez-Marquez J (1999) Prothymosin alpha antisense oligonucleotides induce apoptosis in HL-60 cells. *Cell Death Differ* **6**: 3–5

Roson E, Gallego R, Garcia CT, Heimer EP, Felix AM and Dominguez F (1990*a*) Prothymosin alpha expression is associated to cell division in rat testis. *Histochemistry* **94**: 597–599

Roson E, Garcia CG, Heimer EP, Felix AM and Dominguez F (1990*b*) Cellular distribution of prothymosin alpha and parathymosin in rat thymus and spleen. *J Histochem Cytochem* **38**: 1889–1894

Roson E, Gallego R, Garcia CT, Fraga M, Dominguez F and Beiras A (1993) Evolution of prothymosin alpha and proliferating cell nuclear antigen (PCNA) immunoreactivity through the development of rat ovarian follicles. *Histochem J* **25**: 497–501

Rudolph P, Alm P, Heidebrecht H-J, Bolte H, Ratjen V, Baldetorp B et al (1999) Immunologic proliferation marker Ki-S2 as prognostic indicator for lymph node-negative breast cancer. *J Natl Cancer Inst* **91**: 271–278

Ryazanov AG (1987) $Ca^{2+}$/calmodulin-dependent phosphorylation of elongation factor 2. *FEBS Lett* **214**: 331–334

Sburlati AR, Manrow RE and Berger SL (1991) Prothymosin alpha antisense oligomers inhibit myeloma cell division. *Proc Natl Acad Sci USA* **88**: 253–257

Sigurdsson H, Baldetorp B, Borg A, Dlberg M, Fernö M, Killander D and Olsson H (1990) Indicators of prognosis in node-negative breast cancer. *N Engl J Med* **322**: 1045–1053

Tsitsilonis OE, Bekris E, Voutsas IF, Baxevanis CN, Markopoulos C, Papadopoulou SA, Kontzoglou K, Stoeva S, Gogas J, Voelter W and Papamichail M (1998) The prognostic value of alpha-thymosins in breast cancer. *Anticancer Res* **18**: 1501–1508

Vareli K, Tsolas O and Frangou LM (1996) Regulation of prothymosin alpha during the cell cycle. *Eur J Biochem* **238**: 799–806

Vega FV, Vidal A, Hellman U, Wemstedt C and Dominguez F (1998) Prothymosin alpha stimulates $Ca^{2+}$-dependent phosphorylation of elongation factor 2 in cellular extracts. *J Biol Chem* **273**: 10147–10152

Witzig TE, Ingle JN, Cha SS, Schaid DJ, Tabery RL, Wold LE et al (1994) DNA ploidy and the percentage of cells in S-phase as prognostic factors for women with lymph node negative breast cancer. *Cancer* **74**: 1752–1761

Zalvide JB, Cancio E, Alvarez CV, Regueiro BJ and Dominguez F (1992) Prothymosin alpha mRNA levels are invariant throughout the cell cycle. *J Biol Chem* **267**: 8692–8695

*© 2000 Cancer Research Campaign*
